# Differences in Technical Development and Playing Space in Three UEFA Champions Leagues

**DOI:** 10.3389/fpsyg.2021.695853

**Published:** 2021-08-12

**Authors:** Mario Amatria, Rubén Maneiro, Claudio A. Casal, Sophia Papadopoulou, Hugo Sarmento, Antonio Ardá, Xavier Iglesias, José Luís Losada

**Affiliations:** ^1^Department of Science of Physical Activity and Sport, Pontifical University of Salamanca, Salamanca, Spain; ^2^Department of Science of Physical Activity and Sport, Catholic University of Valencia “San Vicente Mártir”, Valencia, Spain; ^3^Faculty of Physical Education and Sport Science, Aristotle University of Thessaloniki, Thessaloniki, Greece; ^4^Faculdade de Ciências do Desporto e Educação Física, Universidade de Coimbra, Coimbra, Portugal; ^5^Department of Physical and Sports Education, University of A Coruña, A Coruña, Spain; ^6^Institut Nacional d’Educació Física de Catalunya (INEFC), Barcelona, Spain; ^7^Department of Social Psychology and Quantitative Psychology, University of Barcelona, Barcelona, Spain

**Keywords:** soccer, polar coordinates, analysis performance, observational methodology, density of players, orientation change

## Abstract

The analysis of football grows exponentially, with many researchers adopting it as an object of study. The thematic range that addresses it, as well as the different methodologies used, are of a very different nature—physical, psychological, technical, tactical—enriching every day the knowledge, and understanding of the game itself. The objective of this study has been to identify the differences between the different styles of play that lead to becoming the champion of the UEFA Champions League in the last 3 years of the pre-COVID stage, by analyzing the spatial performance developed, the association between the players that make up the different lines of the game system and the analysis of the various technical actions that are developed to carry out the offensive phase of each team. For this, the Observational Methodology and two types of analysis have been used: quantitative, by calculating *X*^2^, and qualitative, by applying the Polar Coordinates technique. The results obtained show the relationship that is established between the different lines that make up the offensive systems of the champion teams of this competition, as well as the relationship of significance that is established between the use of space—width and depth—and the technical means used to achieve success, the goal.

## Introduction

Performance analysis in men’s soccer is experiencing rapid growth in the last decade. Much of the available studies have focused on the offensive phase of the game (Mackenzie and Cushion, 2013; Sarmento et al., 2020). This is common, since it is known that in soccer achieving more goals than the rival is the greatest indicator of success. One of the main characteristics that differentiate soccer from other sports is its low level of goal achievement (Wallace and Norton, 2014).

The performance analysis of the different high-level competitions is allowing us to know how the teams play and what their level of performance is Pratas et al. (2018). The UEFA Champions League is considered the most important team competition at European level. The best teams from each country compete against each other for the title of European team champion.

There are several studies available that analyze this competition from different points of view: the perspective of injuries (Waldén et al., 2005), the physical intensity of the matches (Rave et al., 2018), athletic abilities (Di Salvo et al., 2010), gender differences depending on the distances traveled (Bradley et al., 2014), and even economic circumstances (Espitia-Escuer and García-Cebrián, 2010).

Regarding the measurement of the technical and tactical-strategic components of the game, for 10 years it has been possible to verify an increase in the number of publications. Specifically, the study by Yi et al. (2018), analyzed the technical differences between the different players, finding that the greatest differences were found between defenders and attackers. Specifically, the former achieved better performance in variables related to the pass, and the latter in variables related to the goal and offensive organization. On the other hand, García-Rubio et al. (2015), analyzed the UEFA Champions League competitions between the years 2009 to 2013, they found significant differences in the place of the match, the effect of the first goal and the quality of the rival in this competition, depending on the stage in which it is (group stage or elimination). Along the same lines, the study Yi et al. (2020), also analyze the technical differences depending on the phase in which the teams are, also concluding that the situational variables such as quality of the team and the opponent and the result of the match show variations depending on the technical performance of the players. Likewise, Almeida et al. (2014), also analyze these situational variables and their relationship with the ball’s recovery in 28 UFL games. In their work, they conclude that local and unsuccessful teams defend in more advanced areas of the field, and that the defensive strategies of the best teams involve more intense, organized collective processes that are further from the goal itself.

Regarding the difference between the winning and losing teams in this competition, the study by Lago-Peñas et al. (2011), found that the main variables that discriminate between the winning and losing teams are shots at goal, crosses, ball possession, place of match, and quality of opposition. Tiago et al. (2017), through the use of social networks, analyze 12 matches of the UFC 2015/2016, and observe a negative relationship between the density and the success of offensive action. The reduced density was associated with a greater number of offensive actions, although these have not been successful. Finally, the work of Yi et al. (2019), analyze the differences between teams from different leagues and their performance in the UEFA Champions League, concluding that despite the fact that the differences between teams are small, these could be explained by a cultural aspect, style of play, the characteristics of the players, and the philosophy of the coach.

Given that soccer has evolved tactically at the highest level in recent years (Wallace and Norton, 2014; Bradley et al., 2016), and the constant exchanges of players and coaches produced by the globalization process, it is interesting to investigate the level of performance of the last champions of this competition. Therefore, the objective of this study has been to identify the differences between the different styles of play (Hewitt et al., 2016; Gómez et al., 2018; Lago-Peñas et al., 2018; Castellano and Pic, 2019) that led to the conquest of the UEFA Champions League in the last 3 years of the pre-COVID stage, by analyzing the developed space performance, the association between the players that make up the different lines of the game system, and the analysis of the various technical actions that are developed to carry out the offensive phase of each team.

## Materials and Methods

The present work has made use of the observational methodology (Anguera, 1979), an ideal methodology to develop its potential in sport (Anguera and Hernández-Mendo, 2013). According to Anguera et al. (2011), an observational design has been established: (a) Inter and intra-session follow-up -explanation-; (b) nomothetic -the participating teams-; and (c) multidimensional. The observation has been non-participant and the degree of perceptiveness was total.

### Participants

The participants in this study were the champions of the last three pre-Covid UEFA Champions League, that is, the champions of the 2016–2017 (Real Madrid CF), 2017–2018 (Real Madrid CF), and 2018–2019 (Liverpool FC) competition. All the offensive actions carried out by these teams during the development of their matches are part of this study. In total, 3000 multi-events have been recorded, which have resulted in 387 complex offensive actions (Table 1), made up of all the actions and interventions on the ball that the players perform (simpler actions). Being in turn these individual actions of the players (actions simpler than the final play and that give rise to it), composed of the corresponding multi-events that occur in its development. The collected data belongs to type IV and is, therefore, concurrent and base-time.

**TABLE 1:** Distribution of offensive actions analyzed by team and season.

Season	Team	Plays	Multievents
16–17	Real Madrid CF	100	845
17–18	Real Madrid CF	185	1570
18–19	Liverpool CF	102	586

Of all the offensive actions analyzed by team and season, we are interested in analyzing the depth (Garganta, 1997), the width (Amatria et al., 2019b), and the orientation changes of the different offensive sequences. Likewise, the level of success (Hughes and Bartlett, 2002) achieved in the development of the offensive phase has been analyzed, as well as the level of elaboration and density of the play (Tenga et al., 2009; Sgrò et al., 2016; Amatria et al., 2019a; Table 2).

**TABLE 2:** Density of players in the elaboration of the play.

Number of players involved	Player density
0–1	Non-existent
2–3	Very low
4–5	Low
6–10	Médium
11–15	High
16 or more	Very high

**Number of different players**	**Player density**

0–1	Very low
2–3	Low
4–5	Médium
6–8	High
9–10	Very high
11	maximum

**Number of passes**	**Level of elaboration**

0–1	Non-existent
2–3	Very low
4–5	Low
6–10	Médium
11–15	High
16–20	Very high
21 or more	Maximum

**FIGURE 1:**
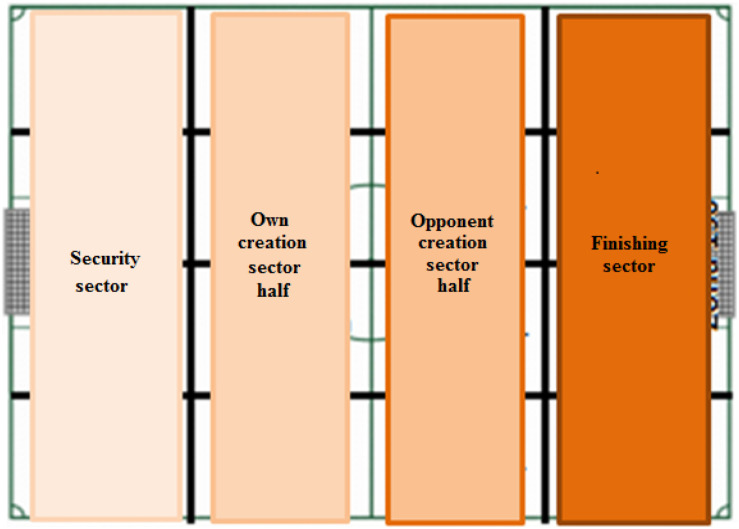
Division into sectors to assess depth. Source: Garganta (1997).

**FIGURE 2:**
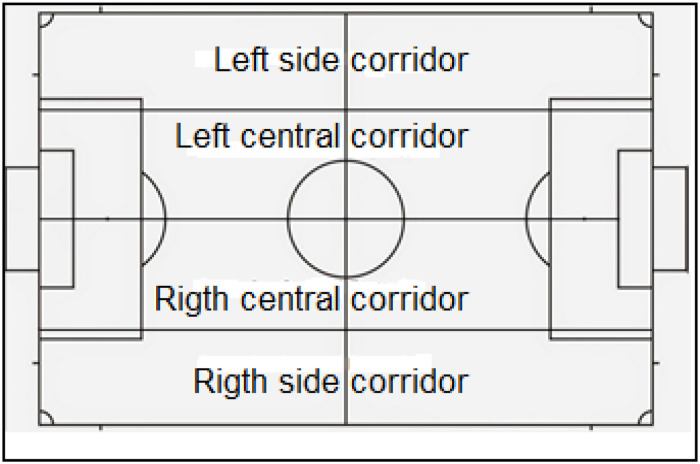
Division into sectors to assess width. Source: Maneiro et al. (2020), adapted from Garganta (1997).

To measure the depth and width of the offensive game, the proposal by Garganta (1997) and Maneiro et al. (2020) has been used (Figures 1, 2).

### Observation Instrument

The observation instrument used has been the one proposed by Maneiro and Amatria (2018), which is a combination of field format and category systems (Anguera et al., 2007); in which all the dimensions of the field format are displayed in category systems by meeting the requirements of exhaustivity and mutual exclusivity (Anguera and Hernández-Mendo, 2013).

### Recording and Coding

The Lince software (Gabin et al., 2012), version 1.2.1, has been used to record the data.

### Data Reliability

The data were recorded by two observers, graduated in Physical Activity and Sports Sciences, both with the title of National Soccer Coach and with extensive experience in the field of observational methodology and the analysis of soccer performance.

In order to determine the reliability -as concordance- of the data obtained from the observation instrument, the GSEQ version 5.1 software was used (Bakeman and Quera, 1995). The Cohen’s Kappa values corresponding to the data packages corresponding to the observations made by both observers have been calculated, comparing the dimensions that make up the observation instrument independently, obtaining a minimum value of κ = 0.95 (Table 3), which guarantees the reliability of the data that support the present work (Fleiss et al., 2003).

**TABLE 3:** Kappa concordance results of the different intra-observer and inter-observer dimensions.

Dimension	Inter-observer Kappa concordance
Ball possession	1
Player	1
Start of action zone	0.96
End of action zone	0.98
Ball contact	0.95
Interruptions	1
Interceptions	1
Completion	1

### Data Analysis

Two types of analysis have been carried out for this study. Regarding quantitative analysis, Pearson’s chi-square statistic (χ^2^) has been used, using the following formula: χ2 = Σk i,j = 1 [(Fij–F^ij)2/F ^ ij]. To obtain the result of this statistic, the SPSS version 20.0 software was used.

On the other hand, taking into account the qualitative analysis, the Polar Coordinate analysis technique has been used to identify the association that occurs between the players of the observed team. This analysis technique is based on Cochran’s Zsum (Cochran, 1954), which in turn takes from the principle that the sum of a number N of independent z scores is normally distributed, with Z = 0 and s = N, so the statistic (where n is the number of lags), according to Sackett (1980), allows us to measure the associative consistency between different behaviors. This technique, developed by Sackett (1980) and improved by Anguera (1997), allows us to identify the relationship of excitation or inhibition between the focal behavior (conditioning behavior), and the rest of the conditioned behaviors (behaviors with which the focal behavior is related). This analysis was carried out both prospectively (+1 to +5) and retrospectively (−1 to −5), obtaining as a result a vector for each behavior related to the focal behavior, with a specific angle and radius. Considering the angle obtained from the analysis, the vector occupies one of the four sectors or quadrants that make up the graphical representation of the Polar Coordinate.

For the representation of the vector map, the R program has been used (Rodríguez-Medina et al., 2019).

## Results

### Study of the Depth of the Game

In reference to the study of game depth, Table 4, there are significant differences in all seasons (2016–2017: *x^2^* = 20.085; gl = 9; *p* < 0.017; 2017–2018: *x^2^* = 20.317; gl = 9; *p* < 0.016; 2018–2019: x2 = 31.824; gl = 9; *p* < 0.001) when analyzing the sector of the start of the offensive action and the sector where it ends. Highlighting, in all seasons, the actions that begin in the definition sector and conclude in the same sector are those with a higher percentage. In this sense, the results obtained in the 2017–2018 season are highlighter, and those plays that have their origin in the sector Creation of the Rival Field and end in the Definition sector have a percentage very close to those that start and end in the same sector, the Definition sector, while it can be seen how there are plays that end in the rest of the sectors, an aspect that does not happen in either of the other two seasons.

**TABLE 4:** Contingency table: Season * Start Sector * Final Sector.

			Final sector			
			Defensive (%)	CP creation (%)	CR creation (%)	Definition (%)
Season 16–17	Start sector	Defensive	8.20	38.80	18.40	34.70
		CP creation	6.50	9.70	29.00	54.80
		CR creation	0.00	0.00	42.90	57.10
		Definition	0.00	0.00	33.30	66.70
Season 17–18	Start sector	Defensive	10.50	36.80	26.30	26.30
		CP creation	0.00	19.40	25.80	54.80
		CR creation	4.80	9.50	19.00	66.70
		Definition	0.00	0.00	30.80	69.20
Season 18–19	Start sector	Defensive	9.20	37.90	21.80	31.00
		CP creation	0.00	23.10	26.90	50.00
		CR creation	0.00	0.00	17.60	82.40
		Definition	0.00	0.00	10.00	90.00

### Study of the Width of the Game

In relation to the study of the depth of the game, Table 5, there are significant differences in the season 18–19 (*x^2^* = 31.824; gl = 9; *p* < 0.000) when analyzing the lane where the offensive action starts and the lane of completion. Highlighting the actions that begin in the Center Right lane and that end in the same lane are those with a lower percentage, the opposite occurring in the rest of the lanes, that is, the plays that reach a higher percentage in each lane are those that end in the same lane, except for the one mentioned.

**TABLE 5:** Contingency table: Season * Start Lane * Final Lane.

			Final lane				
			Banda Iz (%)	Centro Iz (%)	Centro D (%)	Banda D (%)	Z 130 (%)
Season 16–17	Start lane	Left band	20.00	40.00	20.00	0.00	20.00
		Left center	23.10	41.00	17.90	10.30	7.70
		Right center	25.00	17.90	25.00	32.10	0.00
		Right band	8.70	30.40	17.40	34.80	8.70
Season 17–18	Start lane	Left band	36.70	20.00	16.70	23.30	3.30
		Left center	30.80	23.10	19.20	23.10	3.80
		Right center	17.40	17.40	30.40	21.70	13.00
		Right band	20.80	8.30	20.80	41.70	8.30
Season 18–19	Start lane	Left band	40.00	20.00	15.00	15.00	10.00
		Left center	24.10	34.50	17.20	10.30	13.80
		Right center	29.00	25.80	9.70	32.30	3.20
		Right band	4.50	9.10	27.30	54.50	4.50

### Use of Orientation Change

Continuing with the use of width as a means to develop offensive action, the Change of Orientation (CO) has been studied as a key element to carry it out. In this case, significant differences have been detected (*x^2^* = 21.372; gl = 2; *p* < 0.000) between the seasons and the presence of orientation changes in them. Highlighting the high percentage of non-presence of CO in the 18–19 season (Table 6).

**TABLE 6:** Contingency table: Season * Presence of CO.

	CO presence	
	No (%)	Yes (%)
Seas 16–17	73.00	27.00
Seas 17–18	69.90	30.10
Seas 18–19	94.10	5.90

#### Analysis of the Levels of Success of Offensive Actions

Considering the study of the levels of success of the offensive actions that occur in each of the studied seasons, no significant differences have been identified between them. It should be noted in this sense, as in all seasons, that the success rates increase as the level of success is lower, with Level I being the highest level of success and Level IV being the least successful – Table 7.

**TABLE 7:** Contingency table: Season * Level of Success.

	Success level I		Success level II		Success level III		Success level IV	
	GOAL		End Zone 130		End Zone Area		End Zone Def Sec	
	No (%)	Yes (%)	No (%)	Yes (%)	No (%)	Yes (%)	No (%)	Yes (%)
Seas 16–17	96.0	4.0	93.0	7.0	63.0	37.0	54.0	46.0
Seas 17–18	98.1	1.9	93.2	6.8	72.8	27.2	51.5	48.5
Seas 18–19	98.0	2.0	92.2	7.8	66.7	33.3	46.1	53.9

### Game Density

In reference to the analysis of the density of the game, three clearly differentiated aspects have been taken into account.

In the first place, the density of players of the observed team has been studied, later the total density of players who participate in the development of the play has been studied, where the sporadic participation of the opponents through the IOC has been taken into account, and finally, the level of development of the offensive action, understood as the number of times the ball is intervened in the development of the offensive action.

#### Team Player Density

Considering the total number of participants involved in the play by the observed teams, significant differences have been found in the number of players involved in the offensive action (*x^2^* = 38.530; gl = 10; *p* < 0.000) between the seasons analyzed. As a result, a density of players that intervene in the offensive action by the observed team, of a Low level being the one with the highest percentages in the three seasons, followed by a Medium level of density in season 16–17, a High level of density of players of the team observed in the 17–18 season, and a Very Low density level in the 18–19 season (Table 8).

**TABLE 8:** Contingency table: Season * Density of players of the observed team.

	Density_JUG_Eq					
	Very low (%)	Low (%)	Medium (%)	High (%)	Very high (%)	Max. (%)
Seas 16–17	13.0	36.0	21.0	20.0	10.0	0.0
Seas 17–18	8.7	37.9	17.5	24.3	10.7	1.0
Seas 18–19	23.5	56.9	12.7	6.9	0.0	0.0

#### Total Density

Considering the total number of participants involved in the play, no significant differences were found in the number of players involved in the offensive action (*x^2^* = 30.626; gl = 8; *p* < 0.000) between the seasons analyzed. Resulting in a Very Low density, the one with the highest percentages in the three seasons, followed by a Medium level of elaboration in the 16–17 and 17–18 seasons, and a Low level of total density of players in the 18–19 season (Table 9).

**TABLE 9:** Contingency table: Season * Total player density.

	Total_Density				
	Inexistent (%)	Very low (%)	Low (%)	Medium (%)	High (%)
Seas 16–17	11.0	36.0	21.0	31.0	1.0
Seas 17–18	6.8	36.9	17.5	35.0	3.9
Seas 18–19	16.7	52.9	21.6	8.8	0.0

#### Level of Elaboration of the Action

Regarding the study of the level of elaboration of the play, significant differences have been found (*x^2^* = 35.403 gl = 12; *p* < 0.000) in the number of passes made in offensive actions and seasons studied, where the highest percentages of very low production levels are presented in the three seasons, however, in season 17–18 the average production level stands out as the second highest percentage (Table 10).

**TABLE 10:** Contingency table: Season * Elaboration level.

	Elaboration level						
	Inexistent (%)	Very low (%)	Low (%)	Medium (%)	High (%)	Very high (%)	Max. (%)
Seas 16–17	11.0	29.0	23.0	16.0	15.0	2.0	4.0
Seas 17–18	6.8	34.0	14.6	22.3	10.7	5.8	5.8
Seas 18–19	16.7	49.0	18.6	12.7	2.9	0.0	0.0

#### Typology of Contact Developed

In reference to the study of the technical performance developed by the champion teams of the three seasons reflected by the analysis of the different types of contact that the players make on the ball, significant differences are found in all seasons (season 16–17: *x^2^* = 120.458; gl = 35; *p* < 0.000; season 17–18: *x^2^* = 74.213; gl = 35; *p* < 0.000; season 18–19: *x^2^* = 72.663; gl = 35; *p* < 0.000) when analyzing the lines that make up the offensive systems and the type of contact made by the players belonging to the lines that make up the game system. Highlighting, in all seasons, the types of contact C1 and C2 in the different demarcations (Table 11).

**TABLE 11:** Contingency table: Season * Demarcation * Type of contact.

		Contact							
		C1 (%)	C2 (%)	C3 (%)	C4 (%)	C5 (%)	C12 (%)	C23 (%)	C24 (%)
Seas 16–17	PORT	68.40	21.10	5.30	0.00	0.00	5.30	0.00	0.00
	DEF	30.60	51.90	8.90	5.00	2.30	0.40	0.00	0.80
	MED	33.30	38.40	15.30	9.80	1.20	0.80	0.00	1.20
	DEL	28.80	28.80	15.10	5.50	6.80	5.50	5.50	4.10
Seas 17–18	PORT	33.30	42.90	23.80	0.00	0.00	0.00	0.00	0.00
	DEF	28.80	47.20	14.60	1.90	5.10	0.60	0.60	1.30
	MED	28.10	43.50	18.30	4.30	0.70	2.20	2.50	0.40
	DEL	30.00	29.10	24.50	4.50	2.70	2.70	1.80	4.50
Seas 18–19	PORT	34.40	59.40	6.30	0.00	0.00	0.00	0.00	0.00
	DEF	42.80	26.90	19.30	0.00	9.00	0.70	1.40	0.00
	MED	41.20	20.60	23.50	0.00	10.30	2.90	1.50	0.00
	DEL	34.70	21.30	24.00	2.70	12.00	1.30	2.70	1.30

### Polar Coordinates Analysis

In order to make a more clarifying presentation of the results, the results have been structured according to the three seasons analyzed, placing in each of them the demarcation to be studied as the corresponding focal behavior. Thus, through these analyses it is intended to check the predisposition of the players that make up the different lines with their teammates.

#### Results Corresponding to the 2016–2017 Season

**FIGURE 3:**
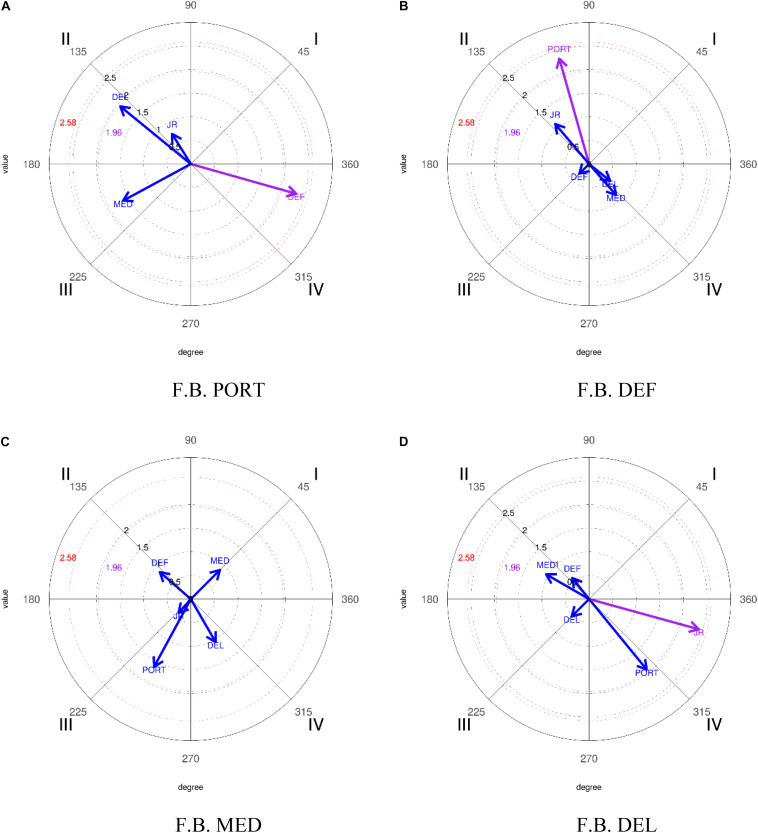
Representation of the behavioral maps establishing the different categories as focal behavior, in relation to the lines that structure the team for the 2016–2017 season. *FB, focal behavior.

In the execution of the analysis, taking PORT as focal behavior and relating it to the rest of the categories that make up this dimension (DEF, CEN, DEL, and JR) the results obtained (Table 12 and Figure 3A) show the criterion category DEF (line of defense) with a radius of 2.32 and an angle of 344.22° in such a way that the vector generated is established in quadrant IV, where the focal behavior activates the appearance of the mating behavior in the prospective plane but not in the retrospective one.

**TABLE 12:** Results of the analysis of polar coordinates for the different focal categories in relation to the rest of the lines that make up the game structure of the 2016–2017 season.

CF	Category	Quadrant	Prospective P	Retrospective P	Ratio	Angle
PORT	DEF	IV	2.23	−0.63	2.32 (*)	344.22
	MED	III	−1.44	−0.78	1.63	208.46
	DEL	II	−1.49	1.22	1.93	140.74
	JR	II	−0.4	0.64	0.75	121.9
DEF	PORT	II	−0.63	2.23	2.32 (*)	105.78
	DEF	III	−0.21	−0.21	0.29	225
	MED	IV	0.57	−0.66	0.87	311.05
	DEL	IV	0.44	−0.35	0.57	321.41
	JR	II	−0.72	0.85	1.11	130.25
MED	PORT	III	−0.78	−1.44	1.63	241.54
	DEF	II	−0.66	0.57	0.87	138.95
	MED	I	0.61	0.61	0.87	45
	DEL	IV	0.52	−0.91	1.05	299.96
	JR	III	−0.25	−0.29	0.39	228.75
DEL	PORT	IV	1.22	−1.49	1.93	309.26
	DEF	II	−0.35	0.44	0.57	128.59
	MED	II	−0.91	0.52	1.05	150.04
	DEL	III	−0.37	−0v37	0.52	225
	JR	IV	2.32	−0.64	2.41 (*)	344.6

Taking DEF as focal behavior and relating it to the rest of the categories that make up this dimension (PORT, DEF, CEN, DEL, and JR), the results obtained (Table 12 and Figure 3B) show the criterion category PORT (goalkeeper line) with a radius of 2.32 and an angle of 105.78° in quadrant II, where the focal behavior inhibits the appearance of the mating behavior in the prospective plane and activates it in the retrospective one.

Finally, establishing DEL as focal behavior and relating it to the rest of the categories that make up this dimension (PORT, DEF, CEN, DEL, and JR), the results obtained (Table 12 and Figure 3D) show the criterion category JR (Rival Player) with a radius of 2.41 and an angle of 344.6° in such a way that the vector generated is established in quadrant IV, where the focal behavior activates the appearance of the mating behavior in the prospective plane but not in the retrospective.

#### Results Corresponding to the 2017–2018 Season

**FIGURE 4:**
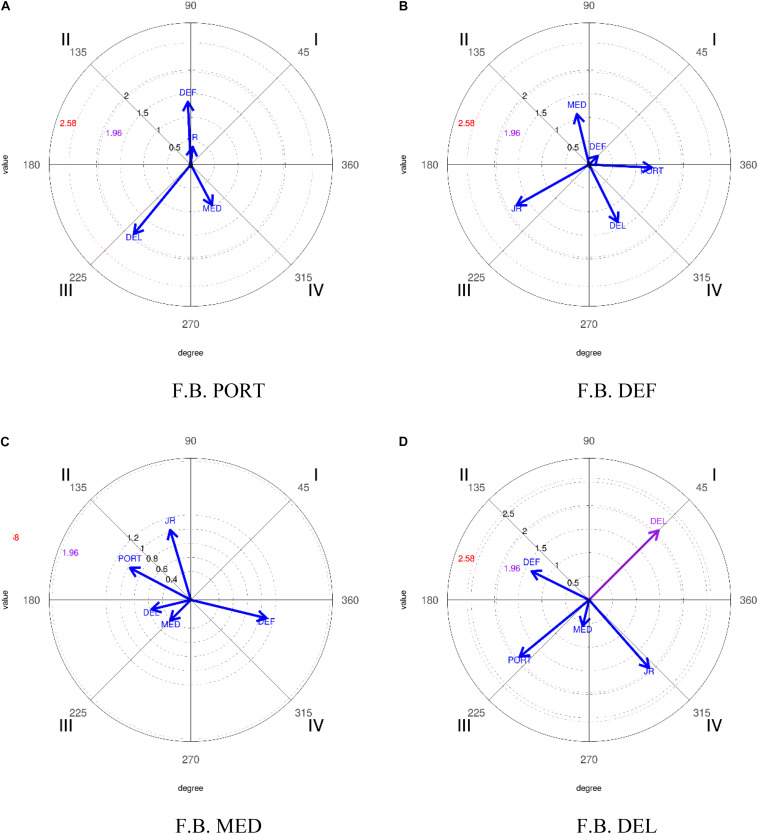
Representation of the behavioral maps establishing the different categories as focal behavior, in relation to the lines that structure the team for the 2017–2018 season. *FB, focal behavior.

In the execution of the analysis, taking as focal behavior the different categories that make up this dimension, no significant results are obtained in any of them except for the DEL category which, relating it to the rest of the categories (PORT, DEF, CEN, DEL, and JR) shows the criteria category DEL (Front Line) in the results obtained (Table 13 and Figure 4) with a radius of 2.08 and an angle of 45° in such a way that the vector generated is established in quadrant I, where the focal behavior activates the appearance of mating behavior at the prospective and retrospective levels.

**TABLE 13:** Results of the analysis of polar coordinates for the different focal categories in relation to the rest of the lines that make up the game structure of the 2017–2018 season.

CF	Category	Quadrant	Prospective P	Retrospective P	Ratio	Angle
PORT	DEF	II	−0.06	1.33	1.33	92.7
	MED	IV	0.45	−0.86	0.97	297.75
	DEL	III	−1.2	−1.47	1.9	230.64
	JR	I	0.04	0.38	0.38	83.96
DEF	PORT	IV	1.33	−0.06	1.33	357.3
	DEF	I	0.18	0.18	0.26	45
	MED	II	−0.26	1.07	1.1	103.59
	DEL	IV	0.61	−1.22	1.36	296.48
	JR	III	−1.54	−0.86	1.76	209.29
MED	PORT	II	−0.86	0.45	0.97	152.25
	DEF	IV	1.07	−0.26	1.1	346.41
	MED	III	−0.29	−0.29	0.41	225
	DEL	III	−0.55	−0.13	0.57	193.6
	JR	II	−0.29	0.98	1.03	106.46
DEL	PORT	III	−1.47	−1.2	1.9	219.36
	DEF	II	−1.22	0.61	1.36	153.52
	MED	III	−0.13	−0.55	0.57	256.4
	DEL	I	1.47	1.47	2.08 (*)	45
	JR	IV	1.27	−1.44	1.92	311.41

#### Results Corresponding to the 2018–2019 Season

**FIGURE 5:**
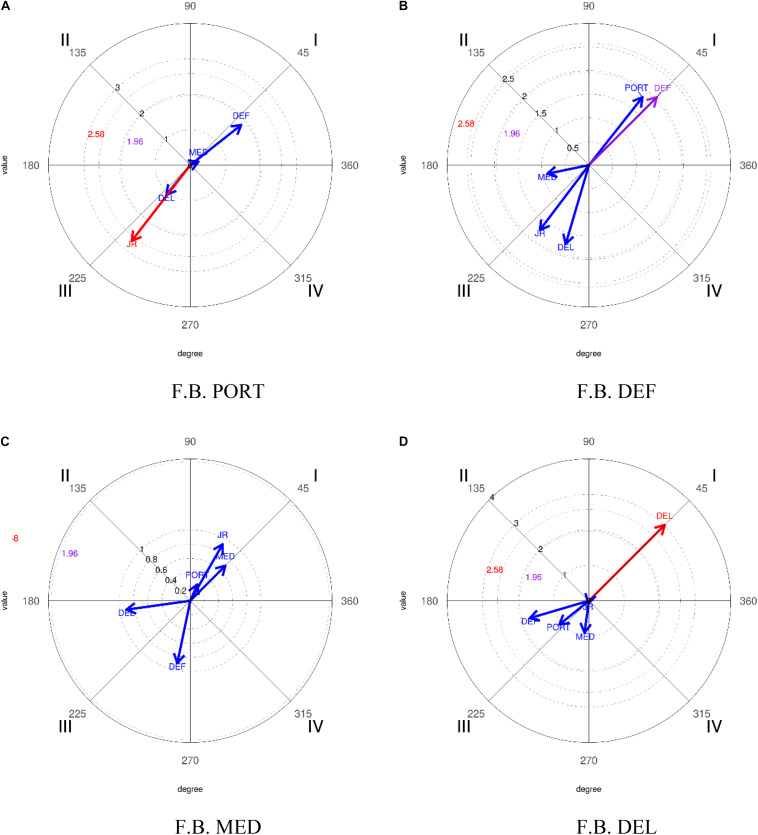
Representation of the behavioral maps establishing the different categories as focal behavior, in relation to the lines that structure the team for the 2018–2019 season. *FB, focal behavior.

In the execution of the analysis, establishing PORT as focal behavior and relating it to the rest of the categories that make up this dimension (DEF, CEN, DEL, and JR), the results obtained (Table 14 and Figure 5A) show the criterion category JR (Rival Player) with a radius of 2.7 and an angle of 232.28° in such a way that the vector generated is established in quadrant III, where the focal behavior inhibits the appearance of the mating behavior in the prospective and the retrospective plane. Taking DEF as focal behavior and relating it to the rest of the categories that make up this dimension (PORT, DEF, CEN, DEL, and JR), shows the criterion category DEF (Line of Defense) in the results obtained (Table 14 and Figure 5B) with a radius of 2.03 and an angle of 45° in quadrant I, where the focal behavior inhibits the appearance of the mating behavior in the prospective plane and activates it in the retrospective one. Finally, establishing DEL as focal behavior and relating it to the rest of the categories that make up this dimension (PORT, DEF, CEN, DEL, and JR), the results obtained (Table 14 and Figure 5D) show the criterion category DEL (Front Line) with a radius of 3.02 and an angle of 45° in quadrant I, where the focal behavior activates the appearance of the mating behavior in the prospective and retrospective plane.

**TABLE 14:** Results of the analysis of polar coordinates for the different focal categories in relation to the rest of the lines that make up the game structure of the 2018–2019 season.

	Category	Quadrant	Prospective P	Retrospective P	Ratio	Angle
PORT	DEF	I	1.44	1.14	1.83	38.27
	MED	I	0.23	0.1	0.25	23.33
	DEL	III	−0.67	−0.83	1.07	231.12
	JR	III	−1.65	−2.13	2.7 (*)	232.28
DEF	PORT	I	1.14	1.44	1.83	51.73
	DEF	I	1.44	1.44	2.03 (*)	45
	MED	III	−0.88	−0.18	0.9	191.76
	DEL	III	−0.49	−1.67	1.74	253.61
	JR	III	−1.04	−1.37	1.72	232.92
MED	PORT	I	0.1	0.23	0.25	66.67
	DEF	III	−0.18	−0.88	0.9	258.24
	MED	I	0.5	0.5	0.7	45
	DEL	III	−0.9	−0.13	0.91	187.93
	JR	I	0.46	0.8	0.92	60.19
DEL	PORT	III	−0.83	−0.67	1.07	218.88
	DEF	III	−1.67	−0.49	1.74	196.39
	MED	III	−0.13	−0.9	0.91	262.07
	DEL	I	2.13	2.13	3.02 (*)	45
	JR	III	−0.02	−0.08	0.08	254.48

## Discussion

This work has been prepared in order to identify the existing differences between the champion teams of the last three Champions Leagues both quantitatively and qualitatively.

Regarding the use of depth in the development of the offensive action, we identified a difference in terms of the achievement of the goal by the three finalists (4, 1.9, and 2% respectively), results that are not in line with those obtained by Kite and Nevill (2017) who obtained results close to 1%. The results obtained in this study are closer to those identified by Amatria et al. (2019a) that amounted to 1.6%, being in all cases higher or much higher. However, when analyzing the real depth of the game, it is observed that more than 50% of the actions that start in any sector other than defensive end in the definition sector, this sector being the one that houses the optimal areas of action (Pollard et al., 2004), with the results being superior to those obtained by Amatria et al. (2019a).

Regarding the development of the amplitude of the game and the use of the spaces that are generated in the side lanes, three different uses for them are identified, highlighting the results of the champion of the 2017–2018 season with results higher than 20% in offensive actions that starting in one side lane finish in the opposite side lane, both from left to right and from right to left (Amatria et al., 2019b). These results show a highly developed use of the amplitude, causing the rival team to perform complete defensive swings (from one side of the field to the other) trying to cause defensive imbalances and taking advantage of favorable numerical-spatial situations. Likewise, it is appreciated how the change of orientation stands as an essential tool to carry out this spatial and numerical use, using this resource in more than 27% of offensive actions, that is, 1 in 4, an aspect that ratifies the Garganta’s theory (1997) and the use of Orientation Changes as a strategic resource to avoid defensive density in the central areas of the field and in the side lanes where the ball is located.

In reference to the number of players, the highest percentages in offensive actions with a low participation of players in the three champions is evidenced. These results are not in line with those obtained by Hughes and Franks (2005) and Lago et al. (2012), although there is a manifest trend toward a high intervention of players (6–8) to reach the shot and send the ball to finishing situations, this type of density—high density—is reflected in the second highest percentages of the first two finals (16–17 and 17–18), not being the case in the final of the 2018–2019 season. The results are in line with those of Tenga et al. (2010) and Maneiro et al. (2019). These results could be explained by the style of play of the finalist teams in terms of the elaboration of the plays (Hewitt et al., 2016), with fast attacks or counterattacks being the ones that occur the most in the three seasons, but in the first two, it alternates with densities that show a more complex development and elaboration of the play, where teams try to play with time and space, and try to overcome a positioned and organized defense. While in the case of the 2018–2019 season, the highest percentages of density of the champion team respond to a game model closely related to the counterattack, reaching, between the Low and Very Low density levels, a percentage close to 70% of the offensive actions. If we identify the teams studied, Real Madrid (champion in the 2016–2017 and 2017–2018 seasons) and Liverpool (champion in the 2018–2019 season), the data corroborate the premise accepted and established by Lago-Peñas and Dellal (2010) which states that successful Spanish teams are the ones with the highest associative percentage, unlike teams of other nationalities (Bradley et al., 2013).

Regarding the analysis of polar coordinates, when analyzing the different lines and their interrelation, in reference to the goalkeeper’s demarcation—PORT—being established as a focal behavior, there are relations of reciprocal activation with the DEF category—the defense line—in quadrant I. This highlights the choice by Real Madrid to develop its offensive action from the most backward positions, the goalkeeper in this case, to develop the action progressively and gradually, in such a way that the opposing team sees itself with the obligation to separate their defensive lines either from each other, or from favorable areas for the completion of the same with a shot or auction.

By taking the defensive line (DEF) as focal behavior, where all the other lines act as mating behaviors, the results are in accordance with what was presented in the previous paragraph. The strong relationship (radius of 2.32) that is established between the defense and the goalkeeper is observed, both prospectively and retrospectively—quadrant I.

Regarding the relationships that are established between the lines, taking the front line as focal behavior and the rest of the lines as mating behaviors, a reciprocal activation—quadrant I—is appreciated between the focal behavior DEL and the JR mating behavior, which manifests the direct interaction between the players who finalize the actions whose main assigned mission is to achieve the goal and the opposing players who try to avoid it by being in field areas of maximum risk for their interests, usually central areas close to the goalkeeper’s area and, consequently, to the goal they must defend.

When performing the same analysis in the 2017–2018 season, we can see that there is no significant relationship between any of the lines established as focal behavior and the rest of the lines as mating behaviors. These results, which at first glance can be misleading, are clarified by the previous statistical analysis. This is because significant results are not appreciated, not because of the lack of relationship between the lines, but because of the variability that is established between them, the permeability and the high degree of connection between the players of the champion team is so high that the relationship between all the lines is similar and consequently there are no significant results. This shows the great capacity for association and the versatility and reading of the players during the development of the game, appearing and participating in the offensive action in areas that, due to their demarcation with stereotyped and pre-established functions, do not correspond which makes the relationship between the players of the different lines constant. Despite this, a relationship of mutual activation—quadrant I—is appreciated, by taking as focal behavior the front line—DEL—and establishing the rest of the lines as conditioned, also compared to itself. This indicates that, although there is great participation by all the players that make up the team in the development of the offensive action, when the ball reaches the front line, they end the action either individually, in which the player receives and ends himself, or by interacting with a partner on the same line, but not with colleagues from farther lines. These data are consistent with what was presented in the previous analysis when studying the density of players, as well as the spatial use of the offensive game by this season’s champion, Real Madrid CF.

Finally, when analyzing the different lines and their interrelation, in reference to the 2018–2019 season, establishing the demarcation of the goalkeeper—PORT—as focal behavior, there are reciprocal activation relationships with the category JR—Rival Player—in the quadrant I. This shows how the Liverpool goalkeeper chooses in his interventions to move the ball away from his goal, generating divided balls, and creating duels where the dispute of the same and the intervention of the rival player occurs more frequently, as it is not an action where the control of the ball is maintained.

By taking the defensive line—DEF—as focal behavior, with the rest of the lines acting as mating behaviors, the results show an association of mutual excitation—quadrant I—with itself. This is indicated by the scarce elaboration of the actions and the underdeveloped game by Liverpool in this final, where the defenders relate to each other, without evaluating the possibility of combining with the other lines of the team to develop their offensive action. The same occurs when taking as focal behavior the front line—DEL—and the rest of the lines as conditioned behaviors, where a behavior of mutual excitement with itself is appreciated, this again indicates that when the front line intervenes, they only relate with themselves and not with the different lines of the team. In this sense, if we relate these results with the previous ones presented in the quantitative analysis, we see that when studying both from the global perspective, a direct game and against it is manifested, where the player belonging to the indicated line takes the ball and is that same player or another on his line who ends the offensive action.

## Conclusion

As a final conclusion of this study, it can be said that there is no single model that brings together the offensive phase of the last three UEFA Champions League champions in their pre-Covid stage. Despite this, the style of play of each of the champion teams can be identified and described both quantitatively and qualitatively, where there are similarities between the first two seasons (16–17 and 17–18) since the champion team and the line-up were the same ones in both years, the Real Madrid CF, where the use of the depth and width of their game is seen, and the use of orientation changes for their development stands out, at a technical level making use mostly of the control and the pass and elaborating the offensive actions with patience elaborating plays that contain between 6 and 10 passes and with an intervention that oscillates between 4 and 5 players. On the other hand, in the last season a much less elaborate and direct style of play is seen, where the depth of the game is very present but the use of the amplitude is not very high, starting and ending the plays in the same lane, an aspect that influences the lack of presence of orientation changes, and developing low or very low levels of processing, where the type of contact that occurs the most is in the C1, a single touch. These marked characteristics respond to the archetype of English football, an aspect that fits in with the champion team of that edition, Liverpool CF.

Finally, it can be stated that there is no single way to achieve victory in a championship of these characteristics and that any path, adjusting to the potential of the players and the circumstances of the match, is valid for achieving victory. However, it is necessary to emphasize that the last champion team was favored by the signaling of a penalty, through which they put themselves ahead on the scoreboard, in the second minute of the game, an aspect that has been able to completely condition the approach of the tactical development of the match by Liverpool FC.

## Future Lines of Research

Future studies go through continuing to investigate the tactical structure of other top-level teams, proposing new tactical alternatives to coaches in their professional performance.

## Limitations

Some of the limitations of the present study are found in the teams analyzed, since two of the three UEFA Champions League champions have been the same team. Another possible limitation can be found in that we do not know the performance of the finals played during the circumstances produced by COVID-19, since confinement and the absence of group training can alter the normal performance of the teams.

## Practical Applications

The practical applications derived from this study include providing tactical alternatives to the different teams participating in international tournaments with the UEFA Champions League format in its Pre-Covid stage. Determining the type and intensity of the relationships that the team establishes between the different lines when it is in possession of the ball will allow the design of specific training tasks, resulting in possible improvements from both an offensive and defensive point of view of the game.

## Data Availability Statement

The raw data supporting the conclusions of this article will be made available by the authors, without undue reservation.

## Author Contributions

MA and RM: data collection and interpretation of the results. JL and XI: methodology. CC and AA: literature review. SP and HS: data analysis. All authors contributed to the article and approved the submitted version.

## Conflict of Interest

The authors declare that the research was conducted in the absence of any commercial or financial relationships that could be construed as a potential conflict of interest.

## Publisher’s Note

All claims expressed in this article are solely those of the authors and do not necessarily represent those of their affiliated organizations, or those of the publisher, the editors and the reviewers. Any product that may be evaluated in this article, or claim that may be made by its manufacturer, is not guaranteed or endorsed by the publisher.
